# Stromal cell-derived factor-1α signals *via* the endothelium to protect the heart against ischaemia-reperfusion injury

**DOI:** 10.1016/j.yjmcc.2019.02.002

**Published:** 2019-03

**Authors:** Daniel I. Bromage, Stasa Taferner, Zhenhe He, Oliver J. Ziff, Derek M. Yellon, Sean M. Davidson

**Affiliations:** The Hatter Cardiovascular Institute, University College London, 67 Chenies Mews, London WC1E 6HX, UK

**Keywords:** Cardioprotection, Endothelial, Cardiomyocyte, CXCR4, SDF-1α, Ischaemia-reperfusion injury

## Abstract

**Aims:**

The chemokine stromal derived factor-1α (SDF-1α) is known to protect the heart acutely from ischaemia-reperfusion injury *via* its cognate receptor, CXCR4. However, the timing and cellular location of this effect, remains controversial.

**Methods and results:**

Wild type male and female mice were subjected to 40 min LAD territory ischaemia *in vivo* and injected with either saline (control) or SDF-1α prior to 2 h reperfusion. Infarct size as a proportion of area at risk was assessed histologically using Evans blue and triphenyltetrazolium chloride. Our results confirm the cardioprotective effect of exogenous SDF-1α in mouse ischaemia-reperfusion injury and, for the first time, show protection when SDF-1α is delivered just prior to reperfusion, which has important therapeutic implications. The role of cell type was examined using the same *in vivo* ischaemia-reperfusion protocol in cardiomyocyte- and endothelial-specific CXCR4-null mice, and by Western blot analysis of endothelial cells treated *in vitro*. These experiments demonstrated that the acute infarct-sparing effect is mediated by endothelial cells, possibly *via* the signalling kinases Erk1/2 and PI3K/Akt. Unexpectedly, cardiomyocyte-specific deletion of CXCR4 was found to be cardioprotective *per se.* RNAseq analysis indicated altered expression of the mitochondrial protein co-enzyme Q10b in these mice.

**Conclusions:**

Administration of SDF-1α is cardioprotective when administered prior to reperfusion and may, therefore, have clinical utility. SDF-1α-CXCR4-mediated cardioprotection from ischaemia-reperfusion injury is contingent on the cellular location of CXCR4 activation. Specifically, cardioprotection is mediated by endothelial signalling, while cardiomyocyte-specific deletion of CXCR4 has an infarct-sparing effect *per se*.

## Highlights

• SDF-1α protects against ischaemia-reperfusion injury, but the mechanism is unknown.
• Exogenous SDF-1α is protective when delivered just prior to reperfusion.
• The acute infarct-sparing effect appears to be mediated by endothelial cells.
• Cardiomyocyte-specific deletion of CXCR4 was found to be cardioprotective *per se*.

## 1 Introduction

Myocardial infarction (MI) is a significant cause or morbidity and mortality. Early reperfusion by primary percutaneous coronary intervention is the most effective strategy for reducing infarct size and improving clinical outcome after ST-elevation myocardial infarction (STEMI) [1,2]. This is significant because infarct size in patients is known to correlate with long-term clinical outcome [3]. However, MI remains a common cause of heart failure and mortality in such patients is high [4,5]. Therefore, cardioprotective strategies to further mitigate the injurious effects of STEMI are paramount. Despite several potential approaches to cardioprotection having been studied, and positive outcomes in several clinical trials, no approaches specifically targeting reperfusion injury are currently used in routine clinical management [6]. Most of these previously investigated approaches have targeted cardiac myocytes directly. Novel approaches, such as those targeting the endothelium, may be more effective, either alone or in combination [7,8].

Stromal derived factor-1α (SDF-1α/CXCL12) is a CXC chemokine that is up-regulated in experimental and clinical studies of MI and regulates chemotaxis of inflammatory and progenitor cells to sites of myocardial injury, thereby beneficially impacting angiogenesis and ventricular remodelling [[9], [10], [11], [12], [13]]. It is a ligand for CXCR4, which is itself upregulated in studies of myocardial infraction and has been used experimentally to target progenitor cells to sites of ischaemic injury [14]. For this reason, SDF-1α-CXCR4 is of considerable interest in ischaemic cardiomyopathy and several studies have demonstrated that prolonged SDF-1α delivery after experimental MI can improve cardiac function [[15], [16], [17], [18], [19]]. Proposed mechanisms include Gα1 dependent activation of phosphoinositide 3 kinase (PI3K), mitogen activated protein kinase (MAPK), and Janus kinase (JAK)-signal transducer and activator of transcription (STAT) signalling, which are also implicated in acute cardioprotection [14].

It is now well established that exogenous SDF-1α confers an acute infarct sparing effect when added prior to ischaemia in pre-clinical models [19]. For example, we recently demonstrated that pre-treatment with exogenous SDF-1α is cardioprotective in both an *ex vivo* rat papillary muscle model as well as isolated human atrial trabeculae muscle [20,21]. However, evidence from mouse models suggests that the timing and cellular locations of SDF-1α-CXCR4 expression and signalling governs its role in protection against and recovery from MI [19]. Using a model of *in vivo* ischaemia-reperfusion injury, we aimed to establish the utility of stimulating SDF-1α-CXCR4 shortly prior to reperfusion, which is of greater therapeutic relevance than treating prior to ischaemia, and to use transgenic mice with CXCR4 deletion restricted to cardiomyocytes or the endothelium to clarify the cellular location of CXCR4 relevant to cardioprotection.

## 2 Methods

### 2.1 Transgenic mice

All use of animals was in accordance with the United Kingdom (Scientific Procedures) Act 1986 (PPL 70/7140) and European Directive 2010/63/EU. A breeding pair of floxed CXCR4 transgenic mice, with insertion of the *loxP* sites around endogenous CXCR4 exon 2 were purchased from The Jackson Laboratory [[22], [23], [24], [25]]. As transgenic homozygote mice lacking CXCR4 die *in utero* [26], these mice were crossed with cardiomyocyte-specific MYH6-MerCreMer mice (The Jackson Laboratory) resulting in a tamoxifen-inducible, cardiomyocyte-specific CXCR4 null bi-transgenic strain on a C57BL/6J background (CM-CXCR4). Endothelial cell CXCR4 null mice (EC-CXCR4) were generated by crossing CXCR4^fl/fl^ transgenic mice with 4-hydroxytamoxifen-inducible endothelial-specific platelet-derived growth factor subunit B (PDGFB)-iCreER^T2^ mice to generate a tamoxifen-inducible endothelium-specific CXCR4^fl/fl^ bi-transgenic strain. Mice were bred to obtain hemizygous (Cre/+) mice and wild type (+/+) littermates for experiments. CXCR4 deletion was induced in mice between 4 and 10 weeks old by administration of tamoxifen as an intraperitoneal bolus daily for 5 consecutive days at a dose of 20 mg/kg [[27], [28], [29]]. Mice were left for 3 weeks after completion of tamoxifen dosing prior to experimentation to ensure loss of CXCR4 protein. Cell-specific Cre-mediated excision of CXCR4 exon 2 following 5 days of tamoxifen administration has been used and characterised previously in myocardial repair experiments. Where appropriate, CXCR4^fl/fl^; Cre^+/+^ mice that were injected with tamoxifen were used as controls and designated EC-CXCR4^WT^ or CM-CXCR4^WT^. CM-CXCR4^+/+^; Cre^+/−^ mice injected with tamoxifen were also used as controls to exclude effects of Cre expression in response to cardiac ischaemia-reperfusion injury. Abbreviations used to describe genotypes are: wild type (WT, +/+); heterozygous (HET, +/−); knockout or mutant (KO, −/−); and homozygous *loxP* site insertion (fl/fl); the Cre transgene is maintained as heterozygous as previously described.

### 2.2 In vivo ischaemia-reperfusion injury

Both male and female mice were used in all *in vivo* experiments for clinical relevance. All *in vivo* data presented is from both sexes and there were no statistically significant differences in the division of sexes between groups. A standard method of *in vivo* IR injury was used [30]. Mice were anesthetised by intraperitoneal injection of 100 mg/kg pentobarbitone sodium, with additional dosage of 17 mg/kg *pro re nata*. Surgery was started once pedal and tail reflexes were abolished and depth of anaesthesia was monitored throughout. Mice underwent orotracheal intubation and positive pressure ventilation without supplementary oxygen. Core body temperature was monitored *via* a rectal temperature sensor and maintained at 36.5 ± 0.5 °C by adjustment of a homeothermic heat mat (Kent Scientific). ECG was recorded throughout using PowerLab 4/25 and Animal Bio Amp coupled to Chart 7 (AD Instruments). A left antero-lateral oblique skin incision was made and the heart exposed *via* a thoracotomy. The LAD was under-run with an 8–0 polypropylene non-absorbable monofilament suture and a snare system used to reversibly occlude of the LAD. Ischaemia, as indicated by ST-segment elevation, was maintained for 40 min before reperfusion was induced by disassembling the snare system. After 2 h of reperfusion, the heart was removed. For *in vivo* experiments, the heart was extracted by transecting the aorta. For *ex vivo* experiments, mice were terminally anesthetised by intraperitoneal injection of 120 mg/kg pentobarbitone sodium at a concentration of 20 mg/ml in 0.9% (*w*/*v*) saline, and 50 IU heparin. Once pedal and tail reflexes were abolished, hearts were extracted and the aorta was cannulated and manually perfused with ice-cold phosphate buffered saline (PBS) until the effluent ran clear. 80 μg/kg or 200 μg/kg rhSDF-1α (R&D Systems) or 0.9% saline vehicle were administered *via* the jugular vein, with these doses based on previous reports in the literature [[31], [32], [33]].

### 2.3 Evaluation of infarct size

The primary endpoint of this *in vivo* model was myocardial infarct size. This is expressed as a percentage of the AAR (IS/AAR), that being the myocardial territory subject to ischaemia during LAD occlusion. The AAR was defined after cannulation of the aorta by re-tightening of the LAD suture and perfusion of 200 μl Evans blue dye. Samples were frozen for 20 min at −80 °C, and stained with triphenyltetrazolium chloride (TTC) for assessment of infarct size on the same day by slicing the heart into five 1 mm sections and incubating them for 20 min in the dark in 1% TTC in phosphate buffer. Following incubation, the sections were fixed in 10% formalin for 24 h before being scanned for analysis by planimetry using ImageJ (version 1.45s, NIH).

### 2.4 qRT-PCR

**Supplementary Fig. 1:**
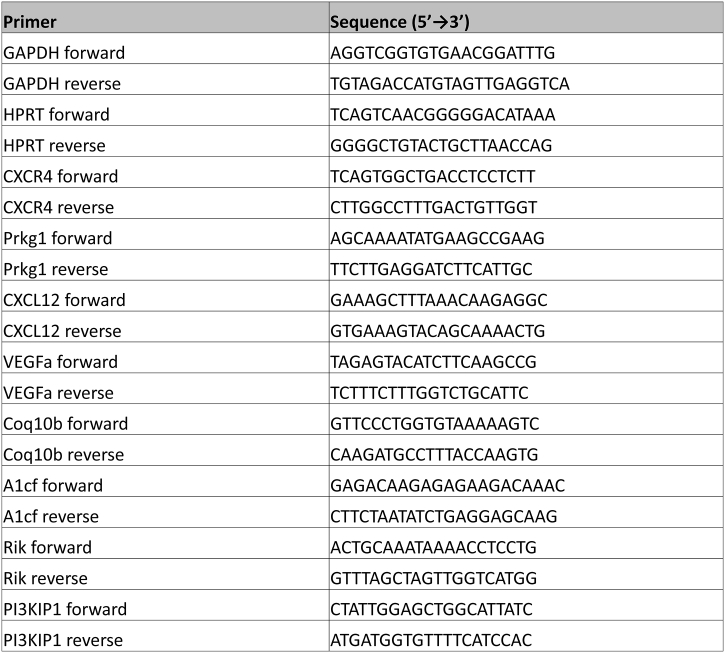
Mouse primer sequences used in qRT-PCR experiments.

mRNA was extracted from a 20–30 mg section of left ventricle (LV) that had been simultaneously disrupted and homogenised by sonication for 10 s using a dedicated kit according to the manufacturer's instructions (RNeasy, Qiagen). 100 ng purified mRNA was converted to first-strand cDNA using the AffinityScript cDNA Synthesis kit (Agilent Technologies), as per the manufacturer's instructions. The QPCR reaction was prepared according to guidelines from Agilent Technologies. Specifically, 12.5 μl of 2× Brilliant II SYBR® Green QPCR Master Mix was mixed with 1 μl each of forward and reverse primer (see Supplementary Fig. 1 for primer sequences), 5.5 μl nuclease-free water and 5 μl template cDNA per reaction. Primers were purchased from Eurofins Genomics. Results were normalised to two reference genes (GAPDH and HPRT) as internal controls using double delta Ct analysis (2^−ΔΔCt^) [34].

### 2.5 HUVECs

5 × 10^4^ human umbilical vein endothelial cells (HUVECs) were seeded per well of a 6-well flat-bottomed plate in 2000 μl M199 medium (Sigma) supplemented with 2% foetal bovine serum (Lonza) until confluence was achieved. rhSDF-1α (Miltenyi Biotec) was added to M199 media to achieve the desired concentration, defined based on previous reports in the literature, for 5 min at 37 °C before lysis buffer was added. Cells were pre-treated with 5 mM of the specific CXCR4 antagonist, AMD3100 (Tocris Bioscience) for 10 min prior to the addition of rhSDF-1α, where relevant. M199 media alone served as a negative control. After treatment, cells were lysed and centrifuged for 10 min at 10,000 rpm and 4 °C. All groups were performed in quadruplicate.

### 2.6 Western blotting

Samples were run using standard sodium dodecyl sulphate-polyacrylamide gel electrophoresis (SDS-PAGE) using 40 μg of protein per sample diluted in lysis buffer. Western transfer was performed to an Immobilon®-FL polyvinylidene difluoride (PVDF) transfer membrane (Merck). Membranes were blocked by incubation in 5% BSA/PBS supplemented with 0.05% Tween® 20 (BSA/PBS-T; ‘blocking buffer’) for 1 h at RT. This was then replaced with the relevant primary antibody diluted 1:1000 5% BSA/PBS-T in blocking buffer (Phospho-p44/42 MAPK (Erk1/2) mouse mAb #9106, Phospho-Akt mouse mAb #4051, from CST). Primary antibodies were incubated at 4 °C overnight. After overnight incubation, non-specifically bound and unbound antibody was removed with six 10 min washes with 0.05% PBS-T followed by addition of the appropriate IR-conjugated secondary antibody (anti-rabbit 800CW or anti-mouse 680LT, Li-Cor) at 1:10,000 dilution in 5% BSA/PBS-T for 1 h at RT. Finally, six 10 min washes in PBS-T were performed, followed by one wash in PBS. Membranes were imaged using the Odyssey® Infrared Imaging System. Protein level was analysed by densitometry using Image Studio™ version 5.0 for Windows (LI-COR), with phosphorylated Akt and Erk being normalised to tubulin loading control for each sample and expressed as arbitrary units (AU). All values are presented as mean AU ± SEM.

### 2.7 Echocardiography

2D transthoracic echocardiography was performed on supine mice using a Vivid *i* ultrasound system with i12S-RS 11 MHz paediatric intra-operative phased-array transducer (GE Healthcare). First, a 2 s cine loop was acquired in the parasternal long-axis view before interrogating the aortic root with pulsed wave (PW) Doppler in the same view. Next, a 2 s cine loop was acquired at papillary muscle level in the parasternal short-axis view and an Motion (M)-mode trace recorded using the papillary muscles as a reference point. After surgery to induce ischaemia-reperfusion injury, a 6-0 braided silk non-absorbable suture (Ethicon) was used to oppose the wound edges when necessary to facilitate echocardiography. All measurements were made by a single observer and were the average of six consecutive cardiac cycles. Analysis was performed offline using EchoPAC (GE Healthcare) according to the experiments' four-digit number to ensure operator blinding. LV end-diastolic and end-systolic internal dimensions (LVIDd and LVIDs, respectively) were measured in M-mode. Temporal resolution was further improved using anatomical M-mode. LVIDd and LVIDs were used to derive fractional shortening (FS) and LV end diastolic volume (EDV) was derived from the long axis end-diastolic dimension and LVIDd. Aortic root velocity-time integral (VTI) was measured using the PW Doppler trace and used to determine stroke volume (SV; μl) [35]. Cardiac output (ml/min) was calculated as a product of SV and heart rate.

### 2.8 RNAseq and analysis

CM-CXCR4^WT^ and CM-CXCR4^KO^ mice were euthanized, the hearts were removed, the aorta cannulated and hearts flushed with PBS. The RNA was extracted from 20 to 30 mg of tissue using RNeasy kit (Qiagen) according to the manufacturer's instructions. The KAPA Stranded mRNA-Seq Kit (Roche) was used to extract mRNA from 100 ng total RNA according to manufacturer's instructions. Strand-specific first strand cDNA was generated using Reverse Transcriptase in the presence of Actinomycin D. The second cDNA strand was synthesised using dUTP in place of dTTP, to mark the second strand. The resultant cDNA was then “A-tailed” at the 3′ end to prevent self-ligation and adapter dimerisation. Truncated adaptors, containing a T overhang, were ligated to the A-Tailed cDNA. Successfully ligated cDNA molecules were then enriched with ~12 cycles of PCR. The primers used extend the adaptor to full length and contain sequences that allow each library to be uniquely identified by way of a sample-specific 6 bp index sequence. Libraries to be multiplexed in the same run were pooled in equimolar quantities, calculated from Qubit and Bioanalyser fragment analysis. Samples were sequenced on the NextSeq 500 instrument (Illumina, San Diego, US) using either a 43 bp or 81 bp paired end run.

Run data were demultiplexed and converted to fastq files using Illumina's bcl2fastq Conversion Software v2.19. Fastq files are pre-processed to remove adapter contamination and poor-quality sequences (trimmomatic v0.36) before being mapped to a suitable reference genome using the spliced aligner STAR (v2.5b). Mapped data was de-duplicated using Picard Tools (v2.7.1), in order to remove reads that are the result of PCR amplification, and remaining reads per transcript were counted by FeatureCounts (v1.4.6p5). Normalisation, modelling and differential expression analysis were then carried out using SARTools (v1.3.2), an integrated QC and DESeq2 BioConductor wrapper.

### 2.9 Immunohistochemistry

Hearts were extracted and perfused as described above, prior to being dehydrated using sequential ethanol steps (25%, 50%, 75%, 100%) and embedded in paraffin. Hearts were sectioned in 5 μm thicknesses and mounted on glass slides. Prior to staining, paraffin-embedded tissues were dewaxed in xylene and rehydrated using ethanol (100%, 75%, 50%, 25%). Samples were steamed in citrate buffer for 10 min and allowed to cool for 20 min at RT. After 3 washes in PBS, sections were incubated with 3% hydrogen peroxide for 10 min to block endogenous peroxidase activity. After a further wash step, sections were incubated with 10% normal rabbit or horse serum diluted in PBS for 1 h (Vector ABC Elite kit; Vector Laboratories), depending on the secondary antibody, and then incubated with the primary antibody at 4 °C overnight with gentle rocking: CD68 mouse mAb (Santa Cruz #sc-70,761), CD45 rat mAb (BD Pharmingen #550539) and Ly6G rat mAb (BD Pharmingen #551459). Secondary only and unstained controls were included. After further washes, the sections were stained using an avidin-biotin-peroxidase complex method (Elite ABC HRP kit, Vector Laboratories). Mast cells were stained for 2–3 min using 0.1% toluidine blue O in 1% NaCl, pH 2.3 (Sigma) The sections were counterstained with hematoxylin for 5 s, washed in running tap water until clear, dehydrated using sequential ethanol steps (as above), cleaned with xylene, and mounted using DPX (Sigma). Images were taken with a 40× objective and a standard light microscope equipped with a digital camera.

### 2.10 Statistics

Sample size was calculated using a two-sided test for the comparison of two means. Based on our previous data, a 20% estimate of effect size, a control infarct size of 40%, an SD of 10%, a significance level of 5% (α = 0.05) and 80% power (β = 0.2) were assumed. This required a minimum sample size of four animals per group.

Results of animal experiments were compared using the Student *t*-test for 2 groups of continuous variables and analysis of variance (ANOVA) and Tukey's Multiple Comparison Test for 3 or more groups. For cell experiments, where the same preparation of cells was used for each treatment, randomized block ANOVA was used as recommended where the values between the groups are correlated [36]. Data is presented as mean ± SEM. Statistical significance was reported if P < 0.05 using the following nomenclature: *P < 0.05, **P < 0.01 and ***P < 0.001. Analyses were performed with GraphPad Prism® version 5.00 for Windows.

## 3 Results

### 3.1 Creation of cell-specific CXCR4^KO^ transgenic mice

Mice with tamoxifen-inducible, endothelial-specific deletion of CXCR4 (EC-CXCR4^KO^), were made by breeding mice harbouring floxed alleles of CXCR4 with mice containing a construct expressing inducible Cre recombinase (iCre) under the control of the PDGFB promoter. Mice with tamoxifen-inducible, cardiomyocyte-specific deletion of CXCR4 (CM-CXCR4^KO^), were made by breeding floxed CXCR4 mice with MerCreMer mice.

cDNA was isolated from CM-CXCR4^WT^ and CM-CXCR4^KO^ mice. By quantitative RT-PCR analysis, CXCR4 expression in CM-CXCR4^KO^ mice was found to be reduced to 37 ± 11% (*N* = 4; *P* < 0.05**)**. In a similar analysis of mice with inducible, endothelial-restricted expression of Cre recombinase, CXCR4 expression was reduced to 38 ± 16% in EC-CXCR4^KO^ mice (*N* = 3; P < 0.05). In cells in which gene expression had been eliminated, residual CXCR4 protein expression is expected to have been eliminated by 3 weeks after the completion of tamoxifen administration.

### 3.2 Exogenous SDF-1α is acutely cardioprotective when administered immediately prior to reperfusion

To evaluate the cardioprotective utility of SDF-1α, mice underwent 40 min of (index) LAD ischaemia and 2 h of reperfusion. As controls, CXCR4^fl/fl^; Cre^+/+^ mice that had not been injected with tamoxifen (*i.e.* phenotypically wild type) were administered with either saline (control), 80 μg/kg SDF-1α or 200 μg/kg SDF-1α by i.v. injection 10 min prior to reperfusion. Infarct size was measured as a proportion of AAR.

**Fig. 1:**
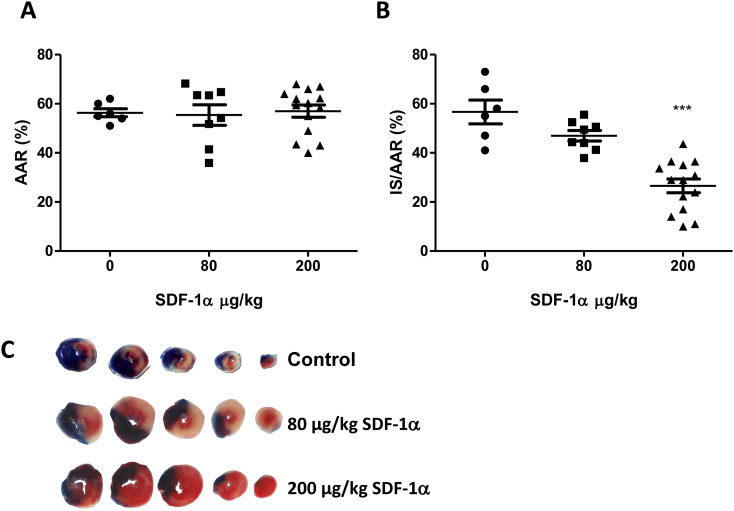
Effect of SDF-1α administration prior to reperfusion on infarct size after *in vivo* ischaemia-reperfusion injury. Phenotypically wild-type mice (CXCR4^fl/fl^ without Cre or tamoxifen) were anaesthetised and the indicated quantities of vehicle or SDF-1α injected prior to 40 min ischaemia and 2 h reperfusion *in vivo;* (A) Analysis of their respective AAR revealed no statistically significant differences; (B) Infarct size (IS) as a proportion of AAR was analysed using Evans Blue and TTC staining. Statistical significance was assessed using one-way ANOVA and Tukey's multiple comparison test, *n* = 6–14, ***P < 0.001 *vs.* 80 μg/kg and control. Data presented as individual hearts with mean ± SEM; (C) Representative scanned transverse heart sections demonstrating Evans Blue area (blue), area at risk (non-blue) and infarct (white). (For interpretation of the references to colour in this figure legend, the reader is referred to the web version of this article.)

CXCR4^fl/fl^ mice were not protected from ischaemia-reperfusion injury. However, SDF-1α significantly reduced infarct size as a proportion of AAR compared to saline vehicle control (*P* < 0.001, Fig. 1) in a dose-dependent manner. This demonstrated that SDF-1α, delivered prior to reperfusion, is acutely cardioprotective in a mouse *in vivo* model of myocardial ischaemia-reperfusion injury. Importantly, it is the first time SDF-1α has been shown to protect when administered immediately prior to reperfusion, thereby illustrating its translational potential. There were no differences in infarct size as a proportion of area at risk according to sex in any of the experiments described (data not shown).

### 3.3 SDF-1α-mediated cardioprotection occurs via endothelial CXCR4 signalling

**Fig. 2:**
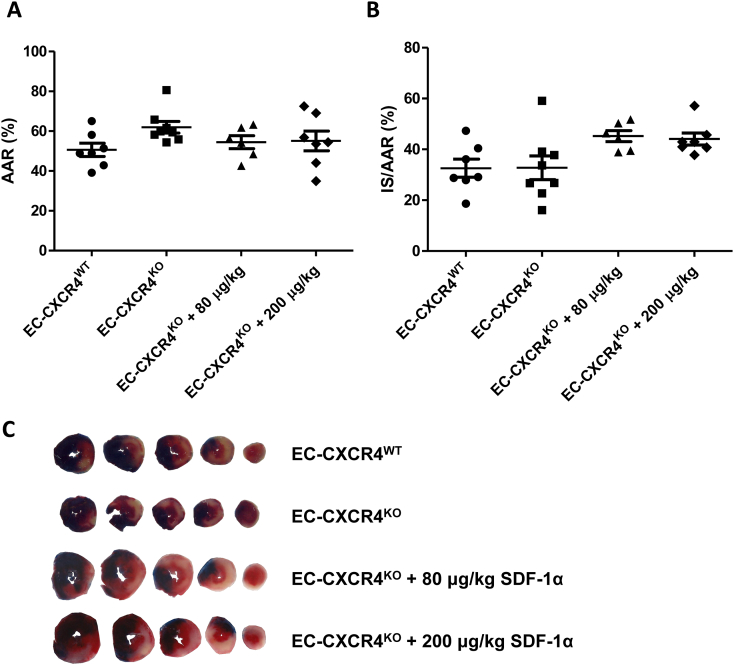
Effect of SDF-1α administration prior to reperfusion on infarct size after *in vivo* ischaemia-reperfusion injury in EC-CXCR4^KO^ mice. EC-CXCR4^WT^ (*i.e.*: CXCR4^fl/fl^; Cre^+/+^) and EC-CXCR4^KO^ (*i.e.*: CXCR4^fl/fl^; Cre^+/−^) mice were injected with tamoxifen as an intraperitoneal bolus daily for 5 consecutive days at a dose of 20 mg/kg. Mice were left for 3 weeks after completion of tamoxifen. Mice were treated with either vehicle or SDF-1α by jugular vein injection and subject to 40 min ischaemia and 2 h reperfusion *in vivo;* (A) Analysis of their respective AAR revealed no statistically significant differences; (B) Infarct size as a proportion of AAR was analysed using Evans Blue and TTC staining and demonstrated no statistically significant differences, *n* = 6–8. Statistical significance was assessed using one-way ANOVA and Tukey's multiple comparison test. Data presented as individual hearts with mean ± SEM; (C) Representative heart sections as per Fig. 1. (For interpretation of the references to colour in this figure legend, the reader is referred to the web version of this article.)

SDF-1α-CXCR4-mediated cardioprotection has been demonstrated in isolated myocytes [33], however since CXCR4 is also present on endothelial cells we investigated if this is important for cardioprotection in a *de novo* experiment. Despite smaller infarct sizes as a proportion of AAR in control (EC-CXCR4^KO^) mice, no difference was observed between EC-CXCR4^KO^ and EC-CXCR4^WT^ groups (Fig. 2), indicating that endothelial CXCR4 deletion is not protective *per se*. Treatment with either 80 μg/kg or 200 μg/kg SDF-1α i.v. 10 min prior to reperfusion did not reduce infarct size in EC-CXCR4^KO^ mice (Fig. 2). This finding demonstrates that protection by exogenous SDF-1α is contingent on endothelial cell CXCR4 activation by SDF-1α.

**Fig. 3:**
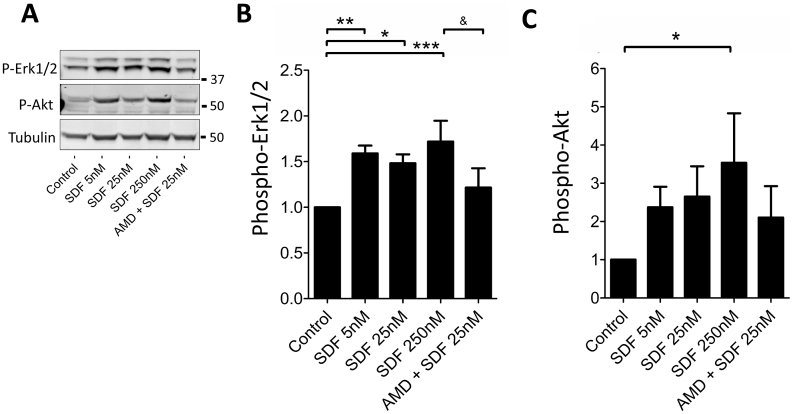
Effect of SDF-1α administration on Phospho-Erk and Phospho-Akt in HUVECs. HUVECs in M199 conditioning media were treated with rhSDF-1α. Further groups were pre-treated 5 mM AMD3100 prior to rhSDF-1α or M199 media alone. After 10 min, cells were washed and analysed by Western blot. Treatment with SDF-1α increased Phospho-Erk relative to tubulin, which was abrogated by pre-treatment with AMD3100. Phospho-Erk and Phospho-Akt were run on the same membrane and normalised to tubulin loading control. Size of standard marker proteins is indicated (kDa). Statistical significance was assessed using repeated measures one-way ANOVA and Tukey's multiple comparison test, *n* = 4, *P < 0.05, **P < 0.01, *** P < 0.001 vs. control; & P < 0.05 vs. 25 nM SDF-1α. Data presented as mean ± SEM.

Treatment of HUVECs with SDF-1α increased phosphorylation of Akt and Erk1/2 in a dose-dependent manner (Fig. 3). Pre-treatment with 5 mM of the specific CXCR4-receptor antagonist AMD3100 abrogated the increased phosphorylation of Erk1/2, although it did not significantly reduce Akt phosphorylation at the tested dose of 25 nM. The reperfusion injury salvage kinase (“RISK”) pathway, including Akt and Erk1/2 [[37], [38], [39], [40], [41]], has been proposed as the mechanism of cardioprotection and is usually assumed to act in cardiomyocytes, but we show here that it can also be activated in endothelial cells, at least *in vitro*.

### 3.4 CM-CXCR4^KO^ transgenic mice are innately resistant against ischaemia-reperfusion injury

**Fig. 4:**
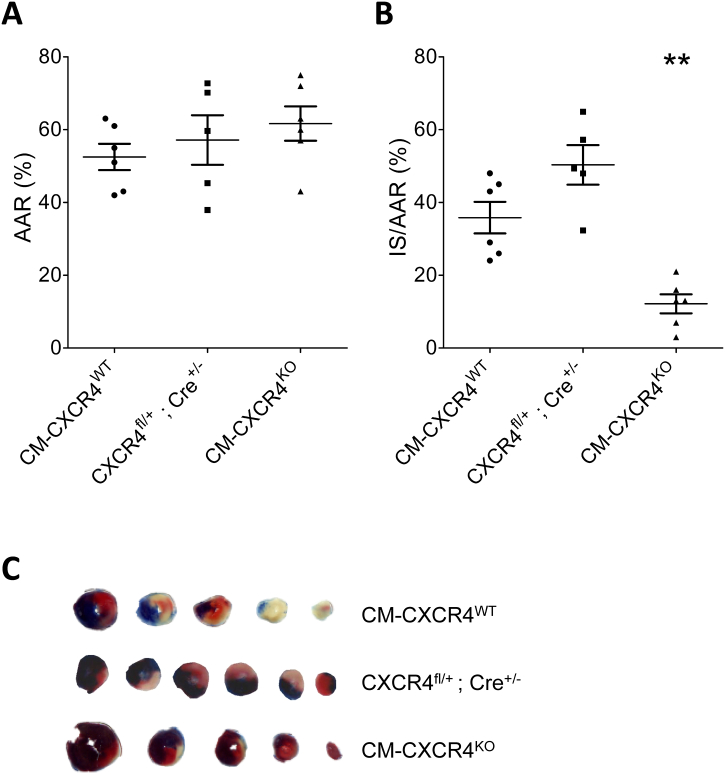
Effect of cardiomyocyte-specific CXCR4 deletion on infarct size prior to *in vivo* ischaemia-reperfusion injury. CM-CXCR4^WT^ (*i.e.*: CXCR4^fl/fl^; Cre^+/+^), CXCR4^fl/+^; Cre^+/−^ and CM-CXCR4^KO^ (*i.e.*: CXCR4^fl/fl^; Cre^+/−^) mice were injected with tamoxifen as an intraperitoneal bolus daily for 5 consecutive days at a dose of 20 mg/kg. Mice were left for 3 weeks after completion of tamoxifen. Mice were anaesthetised and subject to 40 min ischaemia and 2 h reperfusion *in vivo;* (A) Analysis of their respective AAR revealed no statistically significant differences; (B) Infarct size as a proportion of AAR was analysed using Evans Blue and TTC staining and was significantly smaller in CM-CXCR4^KO^ compared to CM-CXCR4^WT^ mice (12.2 ± 2.6% *vs.* 35.8 ± 4.3%). Statistical significance was assessed using one-way ANOVA and Tukey's multiple comparison test, *n* = 6, **P < 0.01 *vs.* CM-CXCR4^WT^. Data presented as mean ± SEM; (C) Representative heart sections as per Fig. 1. (For interpretation of the references to colour in this figure legend, the reader is referred to the web version of this article.)

Infarct size was measured in CM-CXCR4^KO^ and CM-CXCR4^WT^ mice that were subject to *in vivo* myocardial ischaemia-reperfusion injury. Analysis of the areas at risk revealed no significant differences (Fig. 4). Surprisingly, analysis of infarct size demonstrated that, even without administration of SDF-1α, CM-CXCR4^KO^ were innately protected against ischaemia-reperfusion injury, exhibiting a marked reduction in infarct size as a proportion of AAR (*P* < 0.001, Fig. 4). This result precluded further investigation of whether SDF-1α administration could protect these mice against ischaemia-reperfusion injury.

To exclude the possibility that this was a result of the cardiomyocyte-restricted expression of Cre recombinase (which has been shown in some cases to adversely affect cardiac function, myocardial Ca^2+^ handling and energy production [42]), we injected tamoxifen into phenotypically WT mice expressing CM-Cre (CXCR4^fl/+^; Cre^+/−^), and confirmed that they are not protected against ischaemia-reperfusion injury (P=NS *vs.* CM-CXCR4^WT^, Fig. 4).

**Fig. 5:**
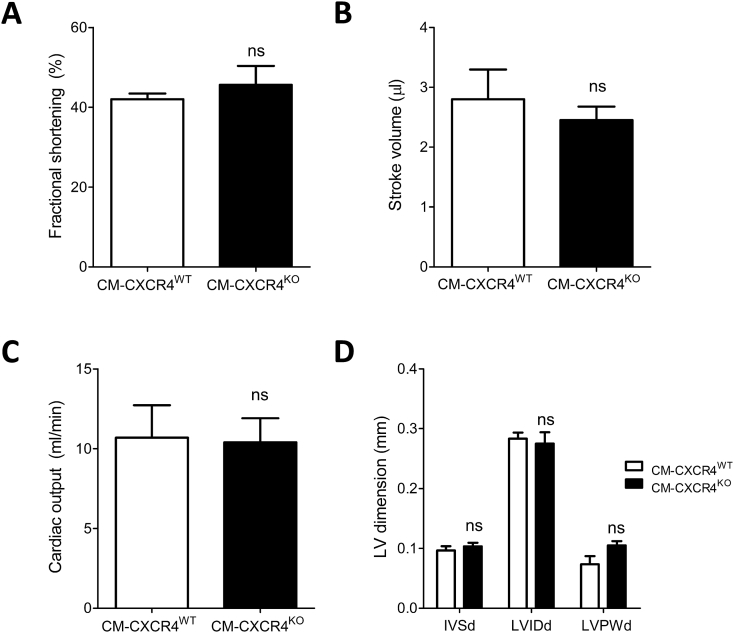
Baseline echocardiographic parameters in CM-CXCR4^WT^ and CM-CXCR4^KO^ mice. CM-CXCR4^WT^ (CXCR4^fl/fl^; Cre^+/+^) and CM-CXCR4^KO^ (CXCR4^fl/fl^; Cre^+/−^) mice were injected with tamoxifen as an intraperitoneal bolus daily for 5 consecutive days at a dose of 20 mg/kg. Mice were left for 3 weeks after completion of tamoxifen. There were no statistically significant differences between CM-CXCR4^WT^ and CM-CXCR4^KO^ groups with respect to (A) fractional shortening, (B) stroke volume, (C) cardiac output or (D) LV dimensions at baseline. Statistical significance was assessed using unpaired *t*-test for A-C and two-way ANOVA with Bonferroni correction for multiple comparisons in D; *n* = 5–6, P=NS.

A second possibility that we considered was that CXCR4 deletion itself altered basal cardiac phenotype, which can affect the response to ischaemia-reperfusion injury. While most cardiomyocyte-specific CXCR4 loss-of-function and gain-of-function studies report a normal cardiac phenotype at baseline [43,44], it has been reported that cardiomyocyte-specific CXCR4 deletion can impair contractility and LV ejection fraction [45,46]. To test the hypothesis that baseline differences in cardiac function may have altered the susceptibility of CM-CXCR4^KO^ mice to myocardial injury, baseline echocardiographic parameters were compared between CM-CXCR4^WT^ and CM-CXCR4^KO^ mice. No differences between CM-CXCR4^WT^ and CM-CXCR4^KO^ mice were identified (Fig. 5). In addition, there was no evidence of cardiac hypertrophy as a result of cardiomyocyte-specific CXCR4 deletion, with no differences in interventricular septum (IVSd), LV internal diameter (LVIDd) and posterior wall (LVPWd) dimensions evident in diastole between groups.

**Supplementary Fig. 2:**
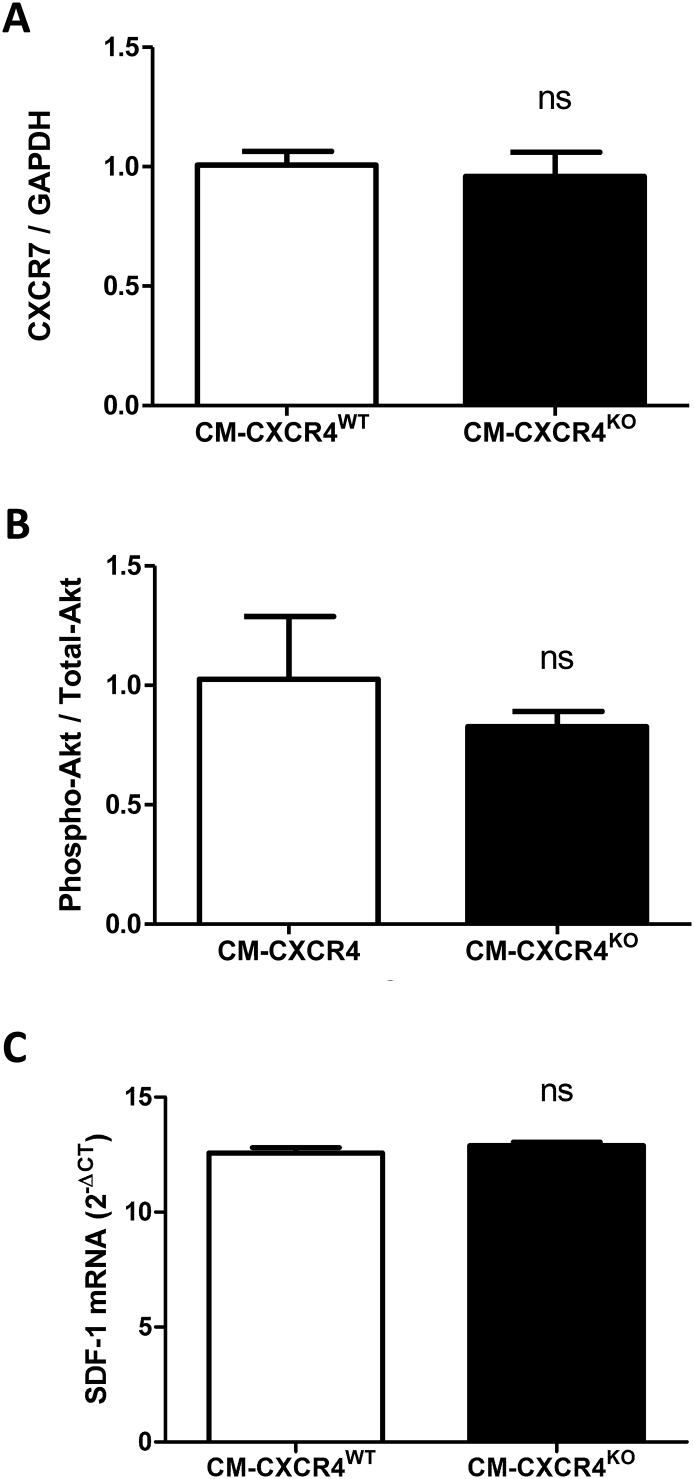
Analysis of whole heart tissue from CM-CXCR4^WT^ and CM-CXCR4^KO^ mice. Western blot analysis demonstrated no difference in CXCR7 (A) or Phospho-Akt / Total-Akt (B) protein expression. QPCR analysis demonstrated no difference in SDF-1α gene expression (C). Statistical significance was assessed using unpaired t-tests, n=4-6. Data presented as mean ± SEM.

Western blot analysis did not reveal any compensatory alteration in cardiomyocyte expression of the alternative SDF-1α receptor CXCR7, and there was no change in levels of the cardioprotective Phospho-Akt / Total-Akt at baseline (Supplementary Fig. 2A–B). Furthermore, there was no difference in SDF-1α mRNA expression in the heart (Supplementary Fig. 2C).

**Supplementary Fig. 3:**
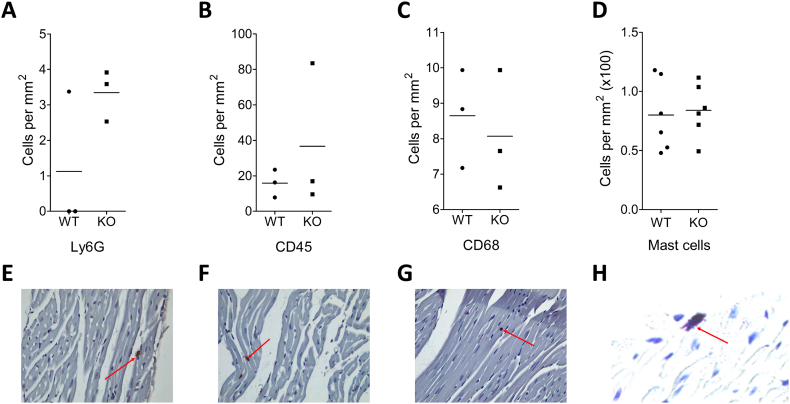
Comparison of baseline inflammatory cells between CM-CXCR4^WT^ and CM-CXCR4^KO^ mice. CM-CXCR4^WT^ (CXCR4^Fl/Fl^; Cre^+/+^) and CM-CXCR4^KO^ (CXCR4^Fl/Fl^; Cre^+/-^) mice were injected with tamoxifen as an intraperitoneal bolus daily for 5 consecutive days at a dose of 20 mg/kg. Mice were left for 3 weeks after completion of tamoxifen; (A-D) 5 μM sections were stained with Ly6G, CD45, CD68 and Toliudine blue for neutrophils, total leukocytes, macrophages and mast cells, respectively. No statistically significant differences between CM-CXCR4^WT^ and CM-CXCR4^KO^ groups were seen at baseline. Statistical significance was assessed using unpaired t-test; n=3-6, P=NS for all comparisons; (E-H) representative sections at x40 magnification with positive staining arrowed.

Finally, we examined the possibility that cardiomyocyte-specific CXCR4 deletion alters inflammatory cell content in the unchallenged heart, based on several previous studies that implicated inflammation in the mechanism of cardioprotection seen with manipulation of the SDF-1α-CXCR4 axis [44,47,48]. However, no significant differences were seen between CXCR4^WT^ and CXCR4^KO^ hearts in the numbers of mast cells (toluidine blue staining), leukocytes (CD45^+^), monocytes/macrophages (CD68^+^) or granulocytes (Ly6G^+^) (Supplementary Fig. 3).

### 3.5 mRNA expression analysis of CM-CXCR4^KO^ mice implicates mitochondrial protein coenzyme Q10b in the mechanism of protection

**Fig. 6:**
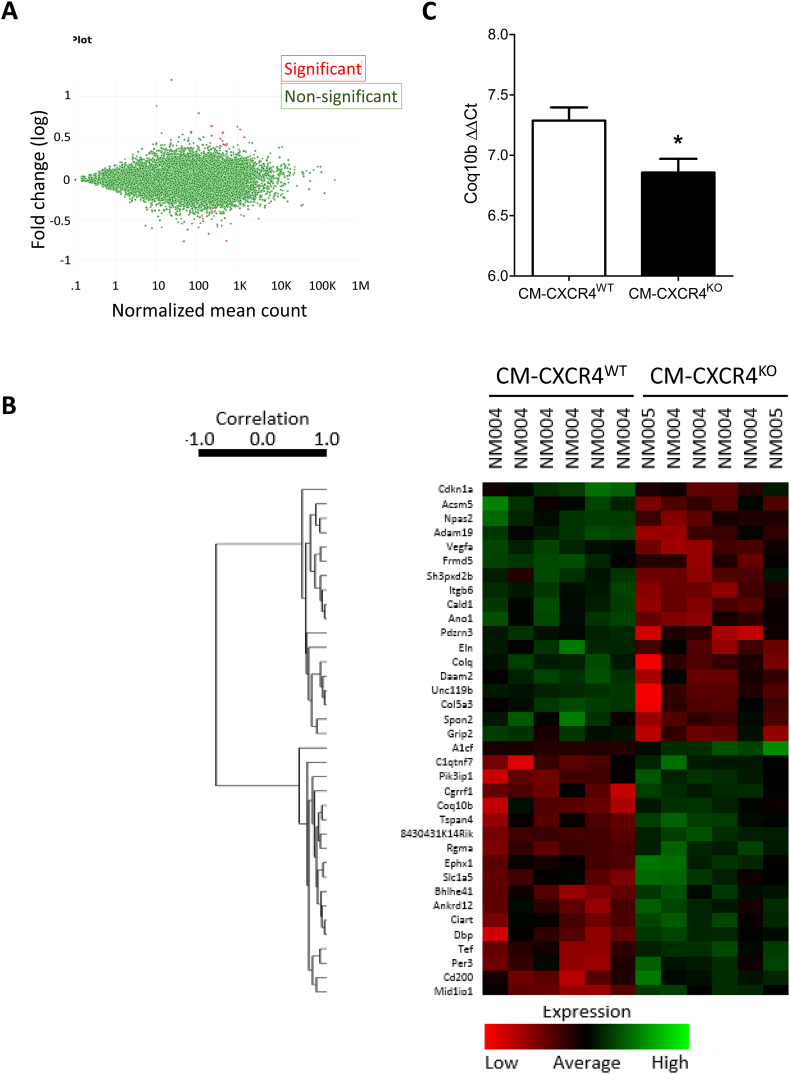
RNAseq analysis comparing mRNA expression in CM-CXCR4^WT^ and CM-CXCR4^KO^ mouse hearts. A) Of ~12K mRNAs detected (green dots), only 37 exhibited a significant difference in expression level (red dots). B) A heat map of genes expressed at significantly different levels. Clustering based on expression patterns demonstrates a clear separation between the CM-CXCR4^WT^ and CM-CXCR4^KO^ hearts (*N* = 6). C) qRT-PCR confirmed an increase in expression of Coq10b in CM-CXCR4^KO^ hearts, resulting in a lower 2^-ΔΔCT^, (*n* = 4, *P* < 0.05 by *t*-test, lower number indicates greater transcript numbers). (For interpretation of the references to colour in this figure legend, the reader is referred to the web version of this article.)

To investigate possible alteration/s in gene expression that might account for the cardioprotective phenotype of the CM-CXCR4^KO^ mice, we performed RNAseq analysis of mRNA expression and compared their expression profile with CM-CXCR4^WT^ mice. This revealed globally very similar expression profiles, but significantly altered expression of 36 mRNAs, 18 of which had increased expression and 18 decreased. (Fig. 6A–B). Several mRNAs with the most consistent expression differences or with published evidence that might be able to account for the protected phenotype were selected for qRT-PCR confirmation of their expression differences. These mRNAs were: Vascular Endothelial Growth Factor A (VEGFA), Phosphoinositide-3-Kinase Interacting Protein 1 (Pik3ip1), Coenzyme Q10B (Coq10b), 8430431K14Rik (a lncRNA) and APOBEC1 Complementation Factor (A1cf). This analysis confirmed a significant increase in the expression only of Coq10b in CM-CXCR4^KO^ hearts (Fig. 6C, *P* = 0.03).

## 4 Discussion

The experiments here were designed firstly to determine whether SDF-1α is cardioprotective when administered acutely at the therapeutically relevant time-point of reperfusion, and secondly to investigate whether protection is mediated by signalling *via* CXCR4 in the endothelium or cardiomyocytes. We confirmed that protection was achievable when 200 μg/kg, but not 80 μg/kg, SDF-1α was administered prior to reperfusion. Cardioprotection by SDF-1α was completely abrogated by the absence of CXCR4 expression in the endothelium. Unexpectedly, when CXCR4 was deleted in cardiomyocytes the mice were rendered innately resistant to ischaemia-reperfusion injury. This may be mediated by the mitochondrial protein co-enzyme Q10b, the mRNA of which was significantly increased in CM-CXCR4^KO^ hearts.

### 4.1 Exogenous SDF-1α is acutely cardioprotective when administered immediately prior to reperfusion

Better functional recovery has been demonstrated after the administration of SDF-1α prior to the onset of ischaemia in an isolated mouse heart model of simulated ischaemia-reperfusion injury [49]. This has similarly been shown in an isolated rat heart model when infused from 10 min prior to reperfusion to 30 min afterwards, in a dose-dependent manner [50]. In the only other *in vivo* model investigating the potential cardioprotective efficacy of SDF-1α, infarct size was significantly reduced when SDF-1α was infused into the LV cavity of C57BL/6 mice prior to subjecting the mice to myocardial ischaemia-reperfusion injury [33]. Importantly, all of these experiments demonstrated a loss of effect of SDF-1α when its cognate receptor, CXCR4, was inhibited with AMD3100. However, no previous studies have administered SDF-1α at the clinically-relevant time point of immediately prior to reperfusion in an *in vivo* model. This timing is significant due to the unpredictable nature of plaque rupture and myocardial infarction in humans. Furthermore, SDF-1α has an estimated plasma half-life of 26 ± 5 min due to proteolysis by various peptidases, including DPP4 [51,52], and it is important to ensure the presence of full-length SDF-1α at the time of reperfusion as it is thought to confer protection by activating protective signalling pathways at this point.

### 4.2 SDF-1α-mediated cardioprotection occurs via endothelial CXCR4 signalling

Lethal cardiomyocyte damage is a major cause of cardiac damage after ischaemia-reperfusion injury. The majority of cardioprotection studies to date have therefore focused on developing strategies to directly protect the cardiomyocytes from such injury. These include most receptor ligands which activate the pro-survival kinase signalling pathways including the RISK pathway (PI3K/Akt and Erk1/2), SAFE pathway (TNFα/JAK/STAT), and PKG pathway in cardiomyocytes [53]. Similarly, SDF-1α-mediated, acute cardioprotection is conventionally thought to be contingent on cardiomyocyte signalling [33]. However, we demonstrate a complete loss of cardioprotection by SDF-1α in mice lacking endothelial CXCR4 (although this deletion was demonstrated in left ventricular tissue rather than isolated endothelial cells). This may be because activation of cardioprotective signalling pathways in the endothelium is subsequently transmitted to the cardiomyocytes. A possible candidate for this intercellular transmission is nitric oxide from nitric oxide synthase, which SDF-1α has been shown to stimulate the production of in cultured endothelial cells [54]. Interestingly, overexpression of SDF-1α in rats has been shown to increase NO production as well as improving arterial patency and inhibiting microsurgical thrombosis in a femoral artery crush model in rats [55]. Similarly, administration of SDF-1α has been seen to increase eNOS activity in other organs such as the kidney, and this was able to preserve microvascular integrity and renal function in chronic kidney disease [56]. Further work is required to determine whether SDF-1α administration increases NO production in the heart.

The specific intracellular mechanism by which SDF-1α induces cardioprotection is not fully defined, but is thought to relate to the activation of the cardioprotective kinases mentioned above [57]. For example, PI3K/Akt and Erk1/2 are implicated both *in vivo* and in direct protection in isolated cardiomyocytes [33,50]. Huang et al. reported that cardioprotection by SDF-1α involved the activation of STAT3, and saw no loss of protection with the PI3K inhibitor LY294002 [49]. Importantly, however, the inhibitors were given prior to ischaemia and not prior to reperfusion (the time-point at which the RISK kinases are defined to act [58]), and hence PI3K/Akt may be integral to mitigating injury specifically at reperfusion. Our data suggest that activation of endothelial signalling is necessary for protection *in vivo*. This possibly occurs *via* the signalling kinases Erk1/2 and PI3K/Akt, although it is a limitation that these are not normalised to total Erk and Akt, and others have similarly shown activation of p-Akt in MVECs treated with SDF-1α [15]. The intracellular mechanism of SDF-1α-mediated cardioprotection is not specifically investigated here as we did not co-treat cells with kinase inhibitors and such inhibitors would affect all cell types in the intact heart, making delineation of the relative contribution of cardiomyocytes and endothelial cells an outstanding question. However, our observation that SDF-1α increases Akt and Erk1/2 expression in endothelial cells, albeit HUVECs rather than primary rodent cells, taken together with studies suggesting that SDF-1α is protective by augmenting endogenous autocrine/paracrine signalling through cardiomyocyte CXCR4 [33], indicates a role for both cardiomyocyte and endothelial SDF-1α-CXCR4 signalling in SDF-1α-mediated cardioprotection.

### 4.3 CM-CXCR4^KO^ transgenic mice are innately resistant against ischaemia-reperfusion injury

The finding of protection in CM-CXCR4^KO^ mice is surprising in view of the literature demonstrating a beneficial role for CXCR4, albeit generally in models of ventricular remodelling. However, many of these studies artificially recruited the SDF-1α-CXCR4 axis, by various means, without necessarily implicating it in the intrinsic response to injury, about which less is known [43,45,59].

Given this finding, it could be hypothesized that SDF-1α administration might increase infarct size in the EC-CXCR4^KO^ mouse, where the protective effects of endothelial CXCR4 signalling are removed. Interestingly, we did see a consistently higher infarct size in the EC-CXCR4^KO^ mice given SDF-1α compared to EC-CXCR4^KO^ controls (Fig. 2B). However, it is difficult to draw conclusions from this since this difference did not reach significance.

In support of our results, adenovirus-mediated over-expression of CXCR4 in rat hearts before myocardial ischaemia-reperfusion has been shown to significantly increase infarct size 24 h later, accompanied by cardiomyocyte apoptosis and worse cardiac function. However, as the analysis was performed at 24 h, there was significant inflammatory cell infiltrate, which may have accounted for the observation [47]. Conversely, Liehn et al. have shown a smaller infarct size in global CXCR4 heterozygous mice (that displayed significantly lower CXCR4 protein expression) subject to permanent LAD ligation compared to WT at 4 weeks, which was also attributed to an attenuated inflammatory response [44]. Our results at 2 h reperfusion suggests that hearts lacking CXCR4 are innately resistant to ischaemia-reperfusion injury, which appears to be independent of baseline inflammatory phenotype, although evaluation of infarct size at further time points is warranted to determine if the changes observed at 2 h are maintained and whether this relates to inflammation. Similarly, a recent study of mice with either inducible or congenital absence of cardiomyocyte-specific CXCR4 subjected to permanent LAD ligation showed no difference in measures of cardiac function or adverse ventricular remodelling compared to CM-CXCR4^WT^ mice at 28 d, suggesting that CXCR4 has no endogenous role in these processes, but infarct size was not specifically measured in this study [60].

It might be inferred that, in view of the protective effect of cardiomyocyte CXCR4 deletion, its activation acutely after MI would be detrimental. However, acute CXCR4 cardiomyocyte activation has been shown not to be detrimental to the cell (and is indeed protective *via* RISK pathways) [33]. Furthermore, studies in isolated rat hearts subjected to regional ischaemia-reperfusion found that AMD3100 was not protective acutely [20,61], nor does it improve cell viability or LDH release from cultured cardiomyocytes subject to hypoxia-reoxygenation *in vitro* [33]. Finally, our group have tested AMD3100 in both human atrial trabeculae and rat papillary muscle and consistently found that acute antagonism of CXCR4 with AMD3100 is not protective [20,21]. This is in contrast to longer term experiments during which AMD3100 may mobilize stem and progenitor cells from the bone marrow that may modify cardiac repair processes [[62], [63], [64]]. Taken together, these data suggest that the observed protection may relate to a cardiac adaptation to loss of CM-CXCR4.

### 4.4 mRNA expression analysis of CM-CXCR4^KO^ mice implicates mitochondrial protein coenzyme Q10b in the mechanism of protection

CoQ10B encodes the mitochondrial protein Coenzyme Q-binding protein COQ10 homolog B which is required for the proper function of coenzyme Q_10_ in the respiratory chain. Its precise role is not known, but it may serve as a chaperone or may be involved in the transport of Q_6_ from its site of synthesis to the catalytic sites of the respiratory complexes [65]. Coenzyme Q_10_ has been suggested to have a dual role in redox signalling and inhibition of death signalling based on experiments in which the enzyme complex was added to rat heart mitochondria [66]. As such, it is possible that altered CoQ10B expression improved mitochondrial function in the transgenic hearts. However, RNAseq data is only hypothesis-generating and confirmation of changes in protein levels, as well as investigating the mechanistic link between CXCR4 deletion and altered CoQ10B expression (including knocking out CoQ10B in mice with cardiomyocyte-specific CXCR4 deletion), is required.

## 5 Conclusions

In conclusion, this study demonstrates that SDF-1α is acutely cardioprotective when administered prior to reperfusion, which occurs *via* endothelial cell signalling. Unexpectedly, CM-CXCR4^KO^ mice were innately resistant to ischaemia-reperfusion injury. Cardiomyocyte CXCR4 expression has previously been implicated in preventing adverse ventricular remodelling and in the development of heart failure after ischaemia-reperfusion injury. It is therefore difficult to understand why CXCR4 deletion results in acute protection in view of the cardioprotective effect of exogenously administered SDF-1α and the evidence for an autocrine/paracrine axis involving the cardiomyocyte. It is likely, given the pleiotropic and opposing effects of the SDF-1α-CXCR4 axis in different aspects of myocardial ischaemia-reperfusion injury that include both acute infarct sparing and longer-scale processes such as angiogenesis and ventricular remodelling, that the timing and cellular location of SDF-1α-CXCR4 activation is key to its effects.

The following are the supplementary data related to this article.

## Funding

This work was supported by grants from the 10.13039/501100000274British Heart Foundation (BHF PG/15/52/31598). DB is an MRC Clinical Research Training Fellow (grant MR/L002043/1). SMD is supported by the Department of Health's NIHR Biomedical Research Centres. ST is support by a 4-year PhD fellowship from the 10.13039/501100000274British Heart Foundation.
